# Increasing sulfate levels show a differential impact on synthetic communities comprising different methanogens and a sulfate reducer

**DOI:** 10.1098/rsif.2019.0129

**Published:** 2019-05-08

**Authors:** Jing Chen, Matthew J. Wade, Jan Dolfing, Orkun S. Soyer

**Affiliations:** 1 School of Life Sciences, University of Warwick, Coventry CV4 7AL, UK; 2 Warwick Integrative Synthetic Biology Centre (WISB), University of Warwick, Coventry CV4 7AL, UK; 3 School of Engineering, Newcastle University, Newcastle NE1 7RU, UK; 4 School of Mathematics and Statistics, McMaster University, Hamilton, Ontario, Canada L8S 4K1

**Keywords:** microbial interaction, anaerobic digestion, community stability, hydrogenotrophic methanogens, acetotrophic methanogens, sulfate-reducing bacteria

## Abstract

Methane-producing microbial communities are of ecological and biotechnological interest. Syntrophic interactions among sulfate reducers and aceto/hydrogenotrophic and obligate hydrogenotrophic methanogens form a key component of these communities, yet, the impact of these different syntrophic routes on methane production and their stability against sulfate availability are not well understood. Here, we construct model synthetic communities using a sulfate reducer and two types of methanogens representing different methanogenesis routes. We find that tri-cultures with both routes increase methane production by almost twofold compared to co-cultures and are stable in the absence of sulfate. With increasing sulfate, system stability and productivity decreases and does so faster in communities with aceto/hydrogenotrophic methanogens despite the continued presence of acetate. We show that this is due to a shift in the metabolism of these methanogens towards co-utilization of hydrogen with acetate. These findings indicate the important role of hydrogen dynamics in the stability and productivity of syntrophic communities.

## 1. Introduction

All studied habitats, ranging from human and animal guts to the soil and ocean, are found to be inhabited by microbial communities composed of hundreds of different species [1]. Interactions among these species ultimately give rise to community-level functions, including metabolic conversions that enable animal and plant nutrition [2,3], and geo-biochemical cycles [4,5]. Understanding the biochemical and physical basis, and the ecological and evolutionary drivers of functional stability in microbial communities is thus a key open challenge in microbial ecology [1]. Achieving a better understanding of these drivers for stable community function can enable prediction of functional stability and collapse thereof [6,7], the design of interference strategies to shift community function [8,9] and the engineering of bespoke ‘synthetic communities’ [10–13].

Towards deciphering ecological and evolutionary drivers of function and functional stability in microbial communities, methanogenic anaerobic digestion (AD) offers an ideal model system, where the production of methane from complex organic substrates can be taken as a proxy for a community function. AD communities are found in many environments including ocean and lake sediments, soil and animal guts and are used in biotechnological re-valuation of organic waste [14]. It is well known that high substrate levels and limited availability of electron acceptors in the AD system can create thermodynamic limitations that can dominate functional stability and community dynamics [15], underpin the emergence and maintenance of diversity in the community [16] and drive evolution of metabolic interactions among different species [17,18]. A key reason for the importance of thermodynamic limitations in AD systems is that it forces a cooperative (i.e. syntrophic) metabolism of organic acids, whereby degradation of these compounds by one group of organisms can only be maintained (i.e. be thermodynamically feasible) by continuous removal of end-products by another [18,19]. This syntrophic degradation can be performed by a range of fermentative microbes including sulfate-reducing bacteria (SRB), while the end-product removal can only be performed by aceto- and hydrogenotrophic methanogens, which specialize in the consumption of acetate and hydrogen, respectively [18,20]. In the case where the syntrophic degradation step is disrupted, acetate and hydrogen can accumulate, leading to further thermodynamic inhibition, as well as acidification, ultimately causing the functional collapse of the AD system [21,22].

A key syntrophic interaction in AD systems is that between SRB and methanogens. This interaction can have a versatile metabolic basis, which has been studied before in controlled co-cultures, but mostly in either the absence or excess presence of sulfate. In the absence of sulfate, for example, certain SRB can ferment organic acids such as lactate and formate to produce H_2_ and acetate, which can be used by aceto- and hydrogenotrophic methanogens [23–26]. In the presence of sulfate, co-cultures of SRB and acetoclastic methanogens show H_2_ consumption and production by these two groups, respectively [27,28]. With sulfate present, it is also possible that SRB can assimilate acetate [29–31]. Based on these documented metabolic interactions, it can be expected that different levels of sulfate can potentially cause either competitive exclusion of methanogens by SRB or cooperative interactions between the two groups. Several studies show that both aceto- and hydrogenotrophic methanogenesis can still coexist with SRB in the presence of significant concentrations of sulfate [32–34] and can persist or adapt to sulfate perturbations [35,36].

It is possible that changes in sulfate levels can affect the stability and type of interaction between SRB and methanogens differently, when different methanogenic groups are involved. Methanogens are distinguished into two major groups through their respiratory and energy-conserving mechanisms, and in particular, whether they contain key respiratory cytochromes or not [20,37,38]. Most hydrogenotrophic methanogens lack cytochromes and are specialized on H_2_, while acetotrophic methanogens encoding cytochromes can grow on low molecular carbons including acetate, methanol and methylamines [20]. Thus, it is possible that hydrogenotrophic methanogens are more susceptible to sulfate perturbation (compared to acetoclastic methanogens) due to competition for H_2_ with SRB. It is, however, also possible that competition for H_2_ with SRB affects those acetoclastic methanogens that maintain an ability for hydrogenotrophic methanogenesis [20,38,39]. These hydrogeno/acetotrophic methanogens are common in the *Methanosarcina* genus [20], and their H_2_ cycling and utilization dynamics are studied in the model organism *Methanosarcina barkeri* [38,40,41]. For obligate acetoclastic methanogens, sulfate perturbation can still be problematic in the presence of sulfate reducers, as some of these can assimilate acetate [29–31].

Given these possible competitive interactions and metabolic versatilities of the involved organisms, it is unclear if and how the productivity and stability of syntrophic interactions between SRB and hydrogenotrophic versus hydrogenotrophic/acetotrophic methanogens differ under different conditions of sulfate perturbation. To answer this question here, we use synthetic communities comprising the model sulfate reducer *D. vulgaris* Hildenborough, the obligate hydrogenotrophic methanogen, *Methanococcus maripaludis* and the hydrogenotrophic/acetotrophic methanogen, *Methanosarcina barkeri*. The latter species is chosen as a representative organism due to its ease of culturability and relevance in a wide range of methanogenic conditions including soils/sediments and AD systems [40,42]. *D. vulgaris* Hildenborough does not mineralize organic carbon substrates and can use lactate to produce acetate as an end-product [43]. We construct synthetic co- and tri-culture communities of these species and evaluate their productivity and stability under sulfate perturbations. We find that tri-culture communities produce twice the amount of methane from lactate compared to co-cultures of the sulfate reducer with a single methanogen. With increasing sulfate availability, however, we find a differential impact on the two methanogenic groups. While *M. maripaludis* was lost from the community at sulfate levels that only allow full respiration of the available lactate, *M. barkeri* was lost readily at lower sulfate levels. This differential stability was also evident at the level of productivity in the tri-culture, where the contribution from *M. barkeri* reduced with increasing sulfate. These results could be explained through mass balance calculations, but only if we assumed a dependency of the *M. barkeri* on hydrogen. We have then verified this assumption experimentally using monocultures. Together, these results show that H_2_-based competition in the presence of strong electron acceptors can influence both aceto- and hydrogenotrophic methanogens, with the former being more prone to be lost from the system as a result. These findings are of high relevance to understand complex, natural AD communities, and to further engineer synthetic communities mimicking their functionality and optimized for specific applications.

## 2. Results

To better understand the functional role and stability of syntrophic interactions between SRB and methanogens in AD communities, we created here a set of synthetic microbial communities composed of two and three species. We used three key species to represent the roles of SRB (*Desulfovibrio vulgaris* Hildenborough*; Dv*), and hydrogenotrophic/acetotrophic (*Methanosarcina barkeri; Mb*) and hydrogenotrophic methanogens (*Methanococcus maripaludis; Mm*). The *Dv*–*Mm* pair has emerged in recent years as a model system to study syntrophic interactions [44] and was recently shown to be enabled by polymorphisms found in *Dv* [45]*. Mb* is one of the most well-studied methanogens capable of hydrogenotrophic/acetotrophic methanogenesis and can be more abundant in AD systems compared with obligate acetotrophic methanogens [40,42,46]. We cultivated these organisms and created relevant synthetic communities composed of one, two and three species (see Material and methods). We initiated replicate synthetic communities using a chemically defined media with lactate (30 mM), as the sole organic carbon source, and cultivated them under different levels of sulfate (see Material and methods). Each constructed community was incubated, and sub-cultured twice, over three-week periods. These conditions mimicked a low-flow, chemostat-like system, while different levels of sulfate mimicked different availability of strong electron acceptors.

### 2.1. All species coexist and community productivity increases in the absence of sulfate

**Figure 1.**
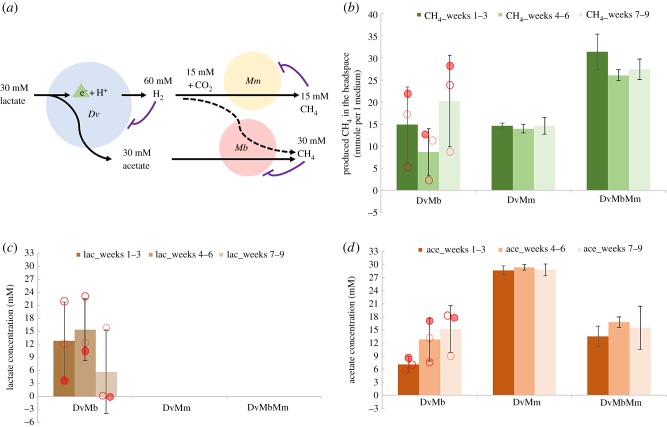
(*a*) Schematic of possible interactions of the three species for converting lactate to methane. The three different species *Desulfovibrio vulgaris* (*Dv*), *Methanococcus maripaludis* (*Mm*) and *Methanosarcina barkeri* (*Mb*) are shown as blue, yellow, and red circles, respectively. The metabolite concentrations shown are those based on the stoichiometries of reactions given in table 2 and using 30 mM initial lactate. Possible thermodynamic inhibitions are indicated by t-ended arrows. The dashed line indicates possible co-utilization of H_2_ by *Mb*. (*b*) Methane produced in the headspace in the absence of sulfate and in the different co- and tri-cultures as indicated on the *x*-axis. Measurements from 5 ml test tube cultures are used to extrapolate to 1 l culture output, so to achieve a better comparison of gas and organic acid data (in mM). (*c*,*d*) Lactate and acetate detected in the liquid phase after 21 days cultivation without sulfate addition. Red dots in (*c*,*d*) refer to the three replicates in the *Dv–Mb* co-cultures. (replicate 1—red hollow circle, replicate 2—dashed red hollow circle and replicate 3—filled red circle). Error bars on (*b*–*d*) are based on three replicates. (Online version in colour.)

The presence of both methanogenesis routes through aceto- and hydrogenotrophic species is expected to increase the productivity in AD communities due to a more complete conversion of the key fermentation products from sulfate reducers (figure 1*a*). We found this expectation to be fulfilled in the absence of sulfate; the synthetic *Dv*–*Mm*–*Mb* tri-culture produced close to twofold more methane compared with the *Dv*–*Mm* and *Dv*–*Mb* co-cultures (figure 1*b*). The tri-culture and the *Dv*–*Mm* co-culture achieved stable methane levels over three sub-cultures, while methane production in the *Dv*–*Mb* co-culture was highly variable. In line with these observations, the tri-culture and the *Dv*–*Mm* system displayed full lactate conversion, while there was significant lactate remaining in one replicate *Dv*–*Mb* system (figure 1*c*). Interestingly, both the tri-culture and the *Dv*–*Mb* co-culture displayed also significant levels of residual acetate (around 7.0–16.0 mM), which was well above the value expected (less than 0.5 mM) from the estimated half saturation coefficient of *Mb* for acetate (*K* = 4.5–5.0 mM) [47,48]. Thus, *Mb* was not able to consume all of the acetate fermented by *Dv* (figure 1*d*)*.* This finding was replicated when we cultivated the cultures under a five-week sub-culturing regime (electronic supplementary material, figure S1), suggesting that lack of expected acetate consumption is not simply due to slow growth of *Mb* on this substrate.

### 2.2. Increased sulfate availability shows a differential impact on the maintenance and productivity of *Mb* versus *Mm*

**Figure 2.**
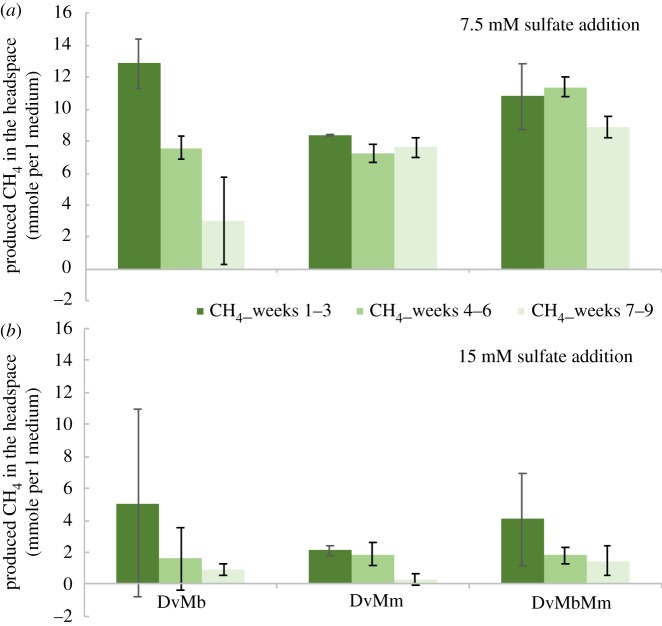
Produced methane in the headspace after 21 days cultivation with 7.5 mM (*a*) and 15 mM (*b*) sulfate addition. The different co- and tri-cultures composed of species *Desulfovibrio vulgaris* (*Dv*), *Methanococcus maripaludis* (*Mm*) and *Methanosarcina barkeri* (*Mb*), as shown on the *x*-axis. Measurements from 5 ml test tube cultures are used to extrapolate to 1 l culture output, so to achieve a better comparison of gas and organic acid data (in mM; see also electronic supplementary material, figure S2). Colours indicate different culturing periods as shown in the legend. Error bars on (*a*,*b*) are based on three replicates. (Online version in colour.)

In order to find out the impact of sulfate availability on the stable coexistence of *Dv* and different methanogens, we further analysed the dynamics of each co-culture and the tri-culture at different sulfate levels. In particular, we cultivated communities in sulfate concentrations that provide either half or full stoichiometric equivalence to lactate; i.e. 7.5 or 15 mM sulfate allowing either half or full respiration of lactate by *Dv* (these conditions are referred to as ‘half-’ and ‘full-sulfate’ from now on). We found that increased sulfate availability immediately impacted the *Dv*–*Mb* co-culture and resulted in a loss of methane production already in half-sulfate treatments (figure 2). The *Dv*–*Mm* co-culture displayed stable coexistence at half-sulfate treatments, but methanogenesis was clearly showing a diminishing trend in the full-sulfate treatment (figure 2). Methanogenesis under increasing sulfate levels in the synthetic tri-culture behaved qualitatively similarly to the *Dv*–*Mm* co-culture, but methane levels in the tri-culture during each culturing period were slightly higher (figure 2).

We found that the impact on methane production by switching from individual co-cultures to a tri-culture also depends on sulfate availability (compare figure 1*b* and figure 2). In particular, we found that going from *Dv*–*Mm* co-cultures to tri-cultures, in the absence of sulfate, increased methane production by almost 100% by the end of the third three-week incubation. Instead, the same comparison shows only a 16.58% increase under the half-sulfate treatment. This suggests that *Mb* populations are either diminishing under the half-sulfate treatment or are not receiving enough acetate. We excluded the latter possibility by measuring lactate and acetate levels for all co-cultures and the tri-culture, and under each sulfate treatment (electronic supplementary material, figure S2). This showed that there are significant levels of acetate in the tri-culture under half-sulfate treatment (as well as full-sulfate treatment), suggesting that the observed smaller increase in productivity (from co- to tri-cultures) compared to the no-sulfate case is not due to acetate limitation.

**Figure 3.**
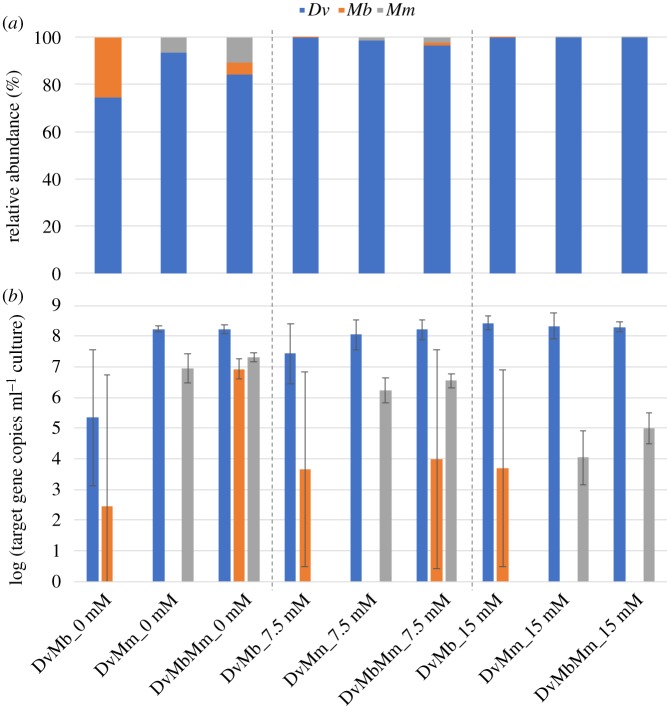
Detected relative gene abundance (*a*) and the absolute abundance of gene copies (*b*) for species *Desulfovibrio vulgaris* Hildenborough (*Dv*), *Methanococcus maripaludis* (*Mm*) and *Methanosarcina barkeri* (*Mb*) in their co- and tri-cultures, as indicated on the *x*-axis. The data for the different species are shown in blue (*Dv*), grey (*Mm*) and orange (*Mb*); 0 mM, 7.5 mM and 15 mM refer to the sulfate concentrations. Results shown are from the end of the third round of 21 days' incubation (weeks 7–9). All results are from three replicates. (Online version in colour.)

To further corroborate these findings, we analysed community stability at the species level by enumerating the different populations using quantitative PCR (qPCR) of the targeted species gene copies at the end of the overall experiment (see Material and methods). In general, *Dv* populations accounted for a large fraction (greater than 80%) of the overall community in all treatments and displayed an increasing trend with sulfate addition (figure 3*a*). An opposite trend is observed for the population sizes of *Mm* and *Mb*, as expected from the observed decrease in methane production. The *Mb* abundance showed high variance in most cases, except for the tri-culture with no sulfate, while *Mm* populations showed an increase in tri-culture (for all distinct sulfate treatments) compared with the same sulfate level co-culture (figure 3*b*). Taken together, these findings show that in the presented system, an increase in community complexity (i.e. extended syntrophic interactions) results in an increased stability of methanogen populations both under sulfate perturbation and without sulfate, and a lower stability of *Mb* populations compared to *Mm*, as sulfate becomes available.

### 2.3. *Mb* populations productivity from acetate shows a significant dependence on H_2_

Why can the acetotrophic *Mb* contribute to methane production under no-sulfate treatment, but not under half- and full-sulfate treatments, even though there is enough acetate available for it to grow? As shown above, *Dv* contributes to a higher fraction of the population with increasing sulfate and can use H_2_, as well as lactate, under this condition [30]. This creates a competitive situation for *Mm*, but possibly also for *Mb*, if it relies also on H_2_ for maintaining its population size. Indeed, we observed H_2_ utilization by *Mb* both in control monocultures, with lactate as the sole carbon source (electronic supplementary material, figure S3), as well as in two replicates in the final sub-culturing of the *Dv*–*Mb* co-cultures under no-sulfate treatment (electronic supplementary material, figure S4).

**Figure 4.**
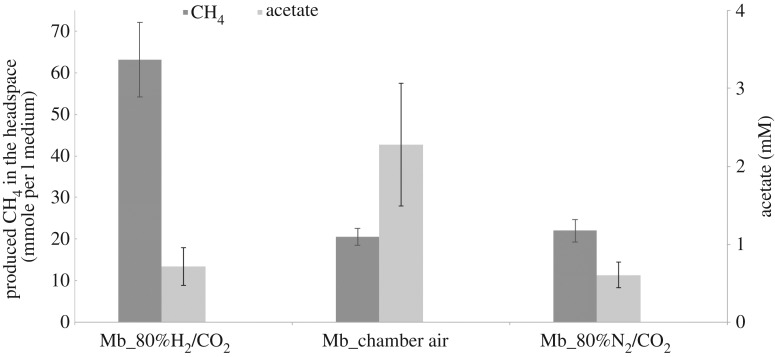
Produced methane in the headspace by *Methanosarcina barkeri* (*Mb*) monocultures (dark grey) and detected residual acetate (light grey) after three weeks of cultivation with 30 mM initial acetate concentration. Note that methane and acetate levels are shown on different *y*-axes. The three sets of results indicate the initial gas composition in the headspace, as shown on the *x*-axis; 80% H_2_ (with 20% CO_2_), 3.14% H_2_ (anaerobic chamber, remaining atmosphere is approx. 89.92% N_2_ and 5.32% CO_2_), and 0% H_2_ (with 80% N_2_ and 20% CO_2_ atmosphere). Error bars are based on three replicates.

These observations, as well as previous indications of H_2_ utilization of *Mb* [20,23,38,39,41], prompted us to more directly test the impact of H_2_ on the growth of *Mb* with acetate, using its monocultures (see Material and methods). These experiments showed that, with acetate provided at 30 mM, increasing H_2_ pressure in the headspace significantly increased *Mb*'s methane production (figure 4). Although most acetate was consumed both in the presence and absence of H_2_, the methane production under the latter condition was only the third of that in the presence of 80% H_2_ in the headspace; 20 mM versus 60 mM methane, respectively. The 1 : 2 stoichiometric relation between acetate and methane in the presence of 80% H_2_ in the headspace, suggests that under this condition, *Mb* uses H_2_ oxidation with acetate reduction, as well as, or in place of, acetotrophic methanogenesis.

### 2.4. Mass balance calculations confirm *Mb*'s use of H_2_ in *Dv*–*Mb* co-cultures

To further evaluate this observation of H_2_ (co)utilization by *Mb* monocultures in the context of the synthetic communities, we performed mass balance calculations using experimental data from the *Dv*–*Mb* co-cultures without sulfate addition (table 1) and the key reactions possible in the system (table 2). Using initial (30 mM) and residual lactate concentrations observed at the end of a three-week cultivation, we derived the observed change in lactate (Δlactate_obs._). We used this value to calculate the theoretical stoichiometric H_2_ and acetate output by *Dv*, assuming full fermentation of lactate by *Dv* (reaction 5 in table 2). We combined these calculated levels with the observed ones (change in headspace H_2_ and residual acetate) to then estimate the theoretical H_2_ and acetate levels that would have been available for *Mb* consumption (H_2*Mb*_ and Acetate*_Mb_*; table 1). For example, in one replicate (row 1 in table 1), we found 20.07 mM residual lactate, indicating 9.93 mM of lactate consumed by *Dv*, resulting in the estimation of acetate and H_2_ production at 9.93 and 19.86 mM, respectively. For this same example replicate, the observed residual acetate was 6.94 mM and headspace H_2_ increased by 2.72 mM from its original level, resulting in the estimation of H_2*Mb*_ and Acetate*_Mb_* at 17.14 and 2.99 mM.

**Table 1.** Observed and calculated substrate levels and per cent consumption and production in the *Desulfovibrio vulgaris* (*Dv*)—*Methanosarcina barkeri* (*Mb*) co-culture without sulfate. Observed lactate levels are obtained from initial and residual levels of this compound in the system, while observed CH_4_ is that measured at the end of three-week cultivation period. Theoretical maximum of H_2_ and acetate that are utilized by *Mb* (columns 4 and 5) is calculated from the theoretical amount available from an assumed full conversion by *Dv* through fermentation (i.e. reaction 5 in table 2), adjusted by the observed residual level of acetate and H_2_ changes compared with its levels in the beginning in the system. *Mb*'s consumption of these substrates and conversion into CH_4_ (column 6) is based on the assumption of it utilizing reactions 1–2, given in table 2 (see Material and methods and main text). The per cent production of CH_4_ as that of possible maximum (column 7) is based on this and the observed CH_4_ (column 3). Finally, *Mb*'s per cent utilization of acetate (column 8) is based on the full conversion of lactate (shown on column 2) and theoretically available to *Mb* (column 5), based on observed residual acetate. The unit for chemicals is mM for organic acids and mmoles per l medium for gases.

co-culture batch (replicate)	observed levels in the system				theoretical maximum used (produced) by *Mb*			production (consumption) as % of possible maximum	
	Δlactate_obs._	acetate	ΔH_2_	CH_4 obs._	H_2 *Mb*_	acetate*_Mb_*	CH_4 calc._	CH_4_	acetate
1 (1)	9.93	6.94	2.72	5.40	17.14	2.99	7.28	74.22	30.11
1 (2)	17.63	5.30	2.50	17.71	32.76	12.33	20.52	86.31	69.94
1 (3)	26.09	8.80	2.85	21.70	49.33	17.29	29.62	73.25	66.27
2 (1)	6.45	7.53	3.25	2.70	9.65	0.00	2.41	111.91	0.00
2 (2)	17.69	13.53	1.92	11.38	33.46	4.16	12.53	90.85	23.52
2 (3)	19.65	17.33	0.63	12.22	38.67	2.32	11.99	101.93	11.81
3 (1)	29.76	18.83	−1.47	23.83	60.99	10.93	26.18	91.03	36.73
3 (2)	13.18	9.07	1.29	8.66	25.07	4.11	10.38	83.45	31.18
3 (3)	30.00	17.77	−4.49	28.31	64.49	12.23	28.35	99.85	40.76

**Table 2.** The compounded, overall growth-supporting reactions considered in the present study. Reactions 1 and 3–5 are primarily thought to be used by *Methanococcus maripaludis* (*Mm*) and *Desulfovibrio vulgaris* (*Dv*), respectively, while reactions 1 and 2 are considered to be possibly (co)used by *Methanosarcina barkeri*. The reaction standard free energy changes at pH 7 (Δ*G*°′) were calculated using tabulated standard Gibbs free energy of formation values for each of the involved compounds [49].

reaction number	equation	Δ*G*°′ (kJ)
1	$4H_{2}+{\mathrm{HCO}}_{3}^{-}+H^{+}\to CH_{4}+3H_{2}O$	−130.7
2	$C_{2}H_{3}O_{2}^{-}+H^{+}\to CO_{2}+CH_{4}$	−35.8
3	$4H_{2}+{\mathrm{SO}}_{4}^{2-}+2H^{+}\to H_{2}S+4H_{2}O$	−157.8
4	$2C_{3}H_{5}O_{3}^{-}+{\mathrm{SO}}_{4}^{2-}\to 2C_{2}H_{3}O_{2}^{-}+2{\mathrm{HCO}}_{3}^{-}+H_{2}S$	−165.8
5	$C_{3}H_{5}O_{3}^{-}+2H_{2}O\to C_{2}H_{3}O_{2}^{-}+{\mathrm{HCO}}_{3}^{-}+2H_{2}+H^{+}$	−4.0

The consumption of these substrates by *Mb* can proceed theoretically through aceto- and hydrogenotrophic pathways (reactions 1 and 2 in table 2), and their possible combination through H_2_ oxidation with acetate reduction. If we use H_2*Mb*_ and Acetate*_Mb_* as given constraints, we can show that the theoretical overall methane output (CH_4calc._) would always be equal to H_2*Mb*_/4 + Acetate*_Mb_* (see Material and methods). We find that the observed methane in the system (CH_4obs._) was almost always below this theoretical maximum ( table 1). There were, however, two cases that result in more methane than theoretically possible, by 1% and 10% more. We find that these two cases present the lowest acetate consumption (no detectable consumption in the second case), and the highest H_2_ consumption, indicating significant H_2_ consumption by *Mb* to produce methane through reaction 1 (and possibly also a combination of reactions 1 and 2). This might have altered *Dv*'s metabolism to shift from acetate fermentation into H_2_ production [45,50] and/or its investment of reductive power into biomass production, which could explain the discrepancy with our theoretical calculation based on reaction 5.

Overall, our results summarized in table 1 show that the methane production in the system cannot be explained solely by acetotrophic methanogenesis but requires involvement from reactions 1 and 2, or their combination. Note that this general conclusion would not be affected by possible investment into biomass by *Dv* or *Mb*, which we neglected in the calculations shown in table 1. Moreover, methane production as a percentage of the theoretical maximum (as calculated above) increases over the course of the three sub-culturing periods, while acetate consumption decreases (table 1). In other words, *Mb* seems to be shifting its metabolism in the presence of *Dv* in a way favouring increasingly H_2_ (co)utilization. This trend, in turn, could explain the instability of *Mb* in the co- and tri-cultures under increasing sulfate conditions, where competition for H_2_ would be higher (due to utilization both by *Dv* and *Mm*).

## 3. Discussion

We have developed here a set of co- and tri-cultures comprising three key functional populations found in AD systems, a sulfate reducer (*Dv*) and aceto- (*Mb*) and hydrogenotrophic (*Mm*) methanogens. These systems allowed us to study the syntrophic interactions among these species under a common ecological perturbation in the form of sulfate availability. Our results showed an increased productivity, in the form of methane production, and high stability, through species coexistence, in the tri-cultures with no sulfate addition. With an increasing availability of sulfate, the shift in *Dv* metabolism towards respiration created a disruption in the methanogen populations, and under non-limiting sulfate concentrations, we found both hydrogenotrophic/acetotrophic and hydrogenotrophic methanogenesis showing a strongly diminishing trend. At limiting levels of sulfate, the disruption to coexistence was also limited, but we found a differentially stronger impact on hydrogenotrophic/acetotrophic populations represented by *Mb*. Experiments on the monoculture of this species verified the strong influence of H_2_ on its growth with acetate, suggesting that its observed instability in tri- and co-cultures could be due to competition with *Dv* and *Mm* for this compound.

Perturbation of methanogenic populations due to competition for H_2_ with SRB has been postulated and studied in several complex communities [32–36]. The presented study, with its well-defined, simplified synthetic communities, provides a direct observation of this competition, and instability of methanogens, in the presence of a sulfate reducer and sulfate as an electron acceptor. More importantly, these synthetic communities reveal that hydrogenotrophic/acetotrophic methanogens are more prone to suffer from such sulfate-inflicted instability despite their ability to use acetate. It would be very interesting to further evaluate this finding in the context of complex AD communities found in nature and in bioreactors. In particular, there is some evidence from the latter systems that hydrogen supplementation can lead to higher methane production [51] which, according to our findings, could be due to a reduction in the competition for H_2_ and enhanced productivity (and possibly growth) of hydrogenotrophic/acetotrophic methanogens.

The synthetic community approach presented here can and should be extended to other combinations of species. In particular, we note that while *Mb* is capable of growth on acetate, there are several other methanogens in nature that seem to have become obligate growers on this substrate, including those from the genus *Methanotrix* (formerly *Methanosaeta*) [37]. It would be very interesting to assess the stability of these obligate acetotrophic methanogens against SRB and hydrogenotrophic methanogens. To this end, a representative species (*Methanosaeta concilii*) from this functional group has already been studied using a synthetic community approach [28], resulting in the identification of both competitive and cooperative interactions with *Dv* and *Mm*. The biochemical underpinning of these interactions, both in that study and the current one, is the flexibility and efficiency of energy conservation mechanisms found in the methanogens [20]. Recent studies have shown that the ability to encode different cytochromes and hydrogenases allows *Mb* (and other methanogens encoding cytochromes) to channel electrons resulting from both the oxidation of one-carbon molecules and H_2_ into the reduction of the key heterodisulfide CoM-S-S-CoB [37]. The resulting electron flow scheme allows *Mb* to perform both aceto- and hydrogenotrophic methanogenesis with higher ATP yield but causes a higher H_2_ pressure requirement for the latter process compared to obligate hydrogenotrophic methanogens [20]. In addition, acetate and one-carbon consumption under this electron flow scheme are suggested to involve H_2_ cycling, whereby H_2_ is generated in the cytosol to then diffuse out of the cell and be re-used at membrane-bound hydrogenases [38]. Both its high H_2_ requirement for hydrogenotrophic methanogenesis, and its possible reliance on H_2_ cycling for aceticlastic methanogenesis, makes *Mb* vulnerable to ecological perturbances as we have shown here.

In this biochemical context, it would be very interesting to see if *Mb* can adapt to co-culturing with *Dv* under a no-sulfate regime and become more tolerant to sulfate-based perturbances. We observe some indication of such possibility, where some of the *Dv–Mb* replicates shifted to significant H_2_ consumption and produced high levels of methane under the no-sulfate treatment. In these cases, we also observe a higher acetate residual (figure 1; electronic supplementary material, figure S4), in line with a previous study of a *Dv*–*Mb* co-culture under sulfate free conditions, where it was suggested that the presence of *Dv* inhibits acetate utilization by *Mb* [23]. Based on our mass balance calculation, however, the observed acetate residual could be explained by a complete switch of *Mb* metabolism to H_2_ oxidation with acetate reduction, as shown in reaction 6. In this scenario, the production of 1 mole of methane only requires 0.5 moles of acetate, fully explaining the observed mass balance in some of the cultures (table 1, figure 1; electronic supplementary material, figure S4). In other words, the acetate consumption by *Mb* would be lower to obtain the same energy yield per reaction if *Mb*'s metabolic pathway follows reaction 6 (as presented in Material and methods, §4.8). Even in the case of half-sulfate treatment, we found high variance in the *Dv–Mb* co-cultures in terms of productivity, indicating the ability of *Mb* to use H_2_. It would be interesting to further evaluate this possibility of *Mb*'s adaptation into a hydrogenotrophic (H_2_/CO_2_) or mixotrophic (H_2_/Acetate) metabolism, and whether the newly identified electron bifurcation mechanisms in hydrogenotrophic methanogenesis pathways of *Mm* [52] could also be present in *Mb* or other hydrogenotrophic/acetotrophic methanogens.

While our combination of *Dv*, *Mm* and *Mb* is not a naturally occurring one and these species have not necessarily undergone coevolution (except throughout these experiments), there is now increasing evidence that the interplay of evolutionary and ecological dynamics is important for the emergence and stability of microbial interactions [53]. For example, recent community coalescence studies find the dominance of entire AD communities over others [54], suggesting co-adaptation among community members being a key drive of productivity and stability. Supporting this view, enriched AD communities are shown to display additional metabolic interactions (particularly auxotrophic interactions) on top of syntrophic interactions [17]. Evolutionary adaptations are also seen in the *Dv*–*Mm* co-culture used here; both species are found to accumulate beneficial mutations when co-evolved in the absence of sulfate [55], and *Dv* populations are found to harbour polymorphisms that directly influence the ability to form a syntrophic interaction with *Mm* [45]. Thus, natural communities might display evolutionary adaptations that render them more resilient to perturbations than our synthetic systems and might display auxiliary interactions on top of the metabolic syntrophies and cross-feeding interactions that we observed here.

Besides their value as experimental hypothesis-generating tools, synthetic communities are also suggested to have potential as specific biotechnological applications [1]. To this end, the co- and tri-cultures presented here can be further expanded with additional functional groups of microbes to attain biotechnologically relevant conversions. It has been suggested, for example, that energy-limited systems presenting thermodynamically driven syntrophic interactions, as well as cross-feeding can provide enhanced productivity compared to monoculture-based bioproduction [56]. Certain chemical conversions and degradations of complex biomaterials, such as cellulose, cannot be achieved by monocultures, and for the evaluation of these compounds, a synthetic community approach, as presented here, will be necessary. Therefore, it would be interesting to expand the tri-culture presented here with primary degraders to allow conversion of complex sugars into methane, as already attempted for cellulose [57]. We advocate the combined use of ecological, evolutionary and engineering approaches to the development and further engineering of such synthetic communities, to achieve robust new biotechnological applications and more representative model ecosystems.

## 4. Material and methods

### 4.1. Strains used

*Desulfovibrio vulgaris* Hildenborough (DSM644, *Dv*-WT), *Methanosarcina barkeri* (DSM800, *Mb*) and *Methanococcus maripaludis* S2 (DSM2067, *Mm*) were originally ordered from the public strain centre DSMZ (www.dsmz.de). The particular *Desulfovibrio vulgaris* Hildenborough strain (referred to as ‘*Dv*’ in this text) used in the present work was previously isolated in our laboratory and contains two key genetic polymorphisms that allow it to grow syntrophically with *Methanococcus maripaludis* without sulfate [45].

### 4.2. Growth media

A defined anaerobic medium, adapted from previous studies [45,50], is used to grow *Dv*, *Mm* and *Mb* in co- and tri-culture. This medium is created by mixing basal salt, trace metal and vitamin stock solutions in appropriate volumes (as explained below). The 1× concentrated basal salt solution was prepared by dissolving the following in 1 l distilled water: 0.19 g K_2_HPO_4_, 2.17 g NaCl, 5.5 g MgCl_2_ × 6H_2_O, 0.14 g CaCl_2_ × 2H_2_O, 0.5 g NH_4_Cl, 0.335 g KCl and 2.5 g naHCO_3_. The 100× concentrated trace element solution was prepared by dissolving the following in 1 l of distilled water and adjusting final solution pH to 7 using HCl and NaOH: 1.5 g nitrilotriacetic acid, 2.48 g MgCl_2_ × 6H_2_O, 0.585 g MnCl_2_ × 4 H_2_O, 1 g NaCl, 0.072 g FeCl_2_ × 4 H_2_O, 0.152 g CoCl_2_ × 6 H_2_O, : 0.1 g CaCl_2_ × 2 H_2_O, 0.085 g ZnCl_2_ × 4 H_2_O, 0.005 g CuCl_2_, 0.01 g AlCl_3_, 0.01 g H_3_BO_3_, 0.01 g Na_2_MoO_4_ × 2 H_2_O, 0.03 g NiCl_2_ × 6 H_2_O, 0.0003 g Na_2_SeO_3_ × 5 H_2_O, 0.008 g Na_2_WO_4_ × 2 H_2_O. The 1000× concentrated vitamin solution was prepared by dissolving the following in 1 l of distilled water: 20 mg biotin, 20 mg folic acid, 100 mg pyridoxin-HCl, 50 mg thiamine-HCl × 2H_2_O, 50 mg riboflavin, 50 mg nicotinic acid, 1 mg vitamin B12, 50 mg d-Ca-panthotenate, 50 mg *p*-aminobenzoic acid, 50 mg lipoic acid. This solution was filter sterilized into a sterile anaerobic serum flask (30 ml in 50 ml flask). All chemicals used are analytic grade or higher (greater than or equal to 98% purity, Sigma-Aldrich, St Louis, MO, USA).

Different carbon and electron acceptor sources were then added to this main media composition, according to the culture used. For the co- and tri-cultures, 30 mM Na-lactate was added as the carbon source, and Na_2_SO_4_ was added at different levels of 0, 7.5 and 15 mM as described in the main text. For *Dv* monocultures, 30 mM Na-lactate and 10 mM Na_2_SO_4_ were added. For *Mb* monocultures, 100 mM Na-acetate was added, and for *Mm* monocultures, 10 mM Na-pyruvate and 682 mM NaCl were added. Furthermore, the *Mm* monoculture headspace was replaced with 2 bar 80%H_2_–20%CO_2_.

All media were prepared anaerobically. First, 10 ml of the 100× trace element stock solution was added to 1× concentrated 1 l basal salt media (with carbon and electron acceptor sources added as explained above). To this, 1 ml Resazurin stock (1 g l^−1^) solution was added, to act as an oxygen indicator. The resulting media was degassed in batches of 200 ml. Each batch was brought to the boiling point in a 500 ml conical flask and then maintained at 80°C under a continuous flow of gas (80% N_2_ + 20% CO_2_) at a flow rate of 0.5 LPM. The gassing line was a blue cannula (0.6 mm ID, Microlance, Beckton Dickinson, Franklin Lakes, NJ, USA) equipped with a sterile filter (Minisart, Sartorius, Göttingen, Germany). After 5 min degassing, 0.2 ml of 1000× vitamin stock solution was added to the (200 ml) medium. To this, 2 ml of cysteine-HCl stock solution (0.2 M) was added to create a reductive environment. The media is then degassed for another hour (at the same flow rate of gas) while being stirred. The removal of oxygen was verified by the Resazurin colour-shift from pink (and occasionally by a redox measurement). All gases (BOC, UK) used for degassing are run through an oxygen scrubber column (Oxisorb, MG Industries, Bad Soden, Germany), to remove any residual oxygen.

After degassing, media were transferred into an anaerobic chamber station (MG 500, Don Whitley). This chamber is maintained according to the manufacture's instruction using N_2_, CO_2_ and H_2_ supplies with an actual gas fraction of 3.14% H_2_ and 5.32% CO_2_, as measured by micro-gas chromatography (GC) (Agilent 490 micro-GC, Agilent Technologies). Before use, any empty culture tubes, rubber stoppers and other tools (i.e. glass baker, electronic dispenser (Eppendorf multipette E3x, Germany) and adaptor (Eppendorf Combtips advanced, Germany)) were degassed for at least 24 h in the anaerobic chamber to exclude any O_2_ contamination. Within the chamber, culture tubes (Hungate anaerobic culture tubes, Chemglass Life Sciences, Vineland, NJ, USA) were filled with 5 ml media. They were then immediately sealed with a blue butyl rubber stopper (Chemglass Life Sciences, Vineland, NJ, USA), transferred out of the chamber, and crimp sealed with aluminium crimp caps (Scientific Laboratory Supplies, Nottingham, UK). Tubes containing the media were autoclaved for 15 min at 121°C in a desktop autoclave (ST 19T, Dixon, Wickford, UK). Before inoculation, a further 0.1 ml of 50× concentrated Na_2_S stock solution was added into the medium to achieve a final concentration of 2 mM Na_2_S. Cultures were then inoculated into such prepared tubes.

### 4.3. Experimental design

**Figure 5.**
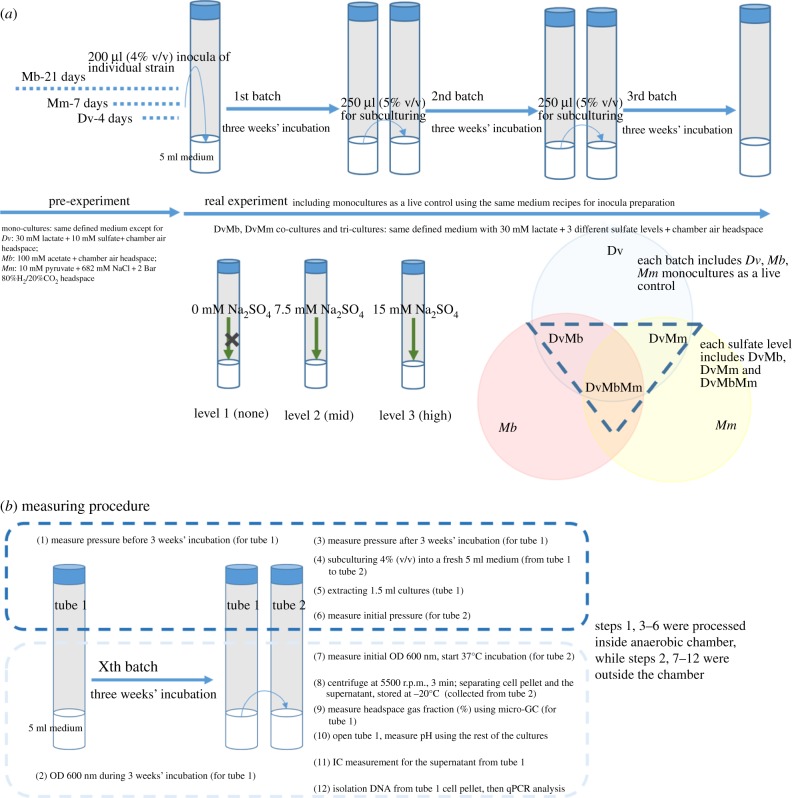
(*a*) A schematic of the design of the main experiment and (*b*) the procedure of the measurement. (Online version in colour.)

Co-cultures of *Dv–Mb* and *Dv–Mm* and tri-cultures of *Dv–Mb–Mm* were constructed as shown in figure 5*a* and tested for the methane production in three batches of cultivation, each of three weeks duration. For co- and tri-culture communities, three treatments of 0, 7.5 and 15 mM sulfate were used, with the latter two treatments corresponding to the half and full theoretical amount required to respire 30 mM lactate (table 1 and figure 5*a*). The cultivations were performed in triplicate, with each set incubated at 37°C for three weeks. The dilution level for sub-culturing was 5% (v/v). In addition, a single round of five weeks' incubation of co-cultures and tri-culture was also conducted. Individual monocultures were also incubated in the same way, to serve as live controls.

The construction of co- and tri-cultures was done using the inoculum from individual monocultures. *Dv*, *Mb* and *Mm* were cultivated until late lag phase for 4, 21 and 7 days, respectively, before inoculation into mixed cultures. For each multi-species culture, 200 µl of individual monocultures are inoculated into 5 ml medium (i.e. 4% v/v).

To test the ability of *Dv*, *Mb* and *Mm* to grow on lactate under the same conditions as co- and tri-cultures, individual monocultures of each strain were incubated with the same medium as those cultures (and under same headspace conditions); 30 mM Na-lactate as carbon source, 7.5 mM Na-sulfate and headspace air fraction same with the anaerobic chamber air.

### 4.4. Gas measurements

Prior to any sampling, overall headspace pressure was measured using a needle pressure gauge (ASHCROFT 310, USA) at the beginning and end of each culturing period (figure 5*b*). The measured pressure was corrected for dead volume of the measurement device, by performing a two-point pressure measurement. The headspace gas composition was analysed using a micro-GC (equipped with a micro thermal conductivity detector and Molsieve 5A and PoraPlot 10 m columns, Agilent 490, Agilent Technologies) at the end of each culturing period. To sample the headspace, a gas-tight glass syringe (Cadence Science, Inc., Italy) was connected to an inert gas sampling syringe valve (Hamilton, USA), a hydrophilic 0.2 µm sterile filter (Sartorius Stedim Biotech GmbH, Gottingen, Germany) and a 23 G sterile needle (Becton Dickinson, S.A., Fraga, Spain). At first, 1 ml gas was extracted from the headspace for filling any dead volume in the sampling system. Then, 2 ml gas was extracted and supplied to micro-GC sampling loop, equipped with a Genie 170 membrane separator (A + Corporation, LLC, LA, USA) to exclude any water contamination. The running parameters for micro-GC analysis were: 100°C column temperature, initial column pressure of 175 kPa (static pressure mode) and 100°C injector temperature for two channels. The injection time for the Molsieve column was 40 ms with 9 s backflush time, and that for the PoraPlot column was 100 ms without backflush.

### 4.5. Culture sampling and pH measurement

At the end of every three weeks culturing period, 1.5 ml culture was extracted using a 1 ml syringe inside the anaerobic chamber and centrifuged at 5500 rpm for 3 min. The biomass and supernatant were separated and stored at −20°C for further DNA extraction and ion chromatography (IC) analyses. After sampling, culture tubes were opened and the residual culture (approx. 3 ml) was pooled out for pH measurement (Mettler Toledo M300, Columbus, Ohio, USA).

### 4.6. Optical density and metabolite measurements

Optical density (OD) of the cultures at 600 nm was measured on a daily basis using a spectrophotometer (Spectronic 200E, Thermo Scientific). Each tube was vortexed for 5 s before each OD measurement using a vortex mixer (Stuart SA8, Stone, UK).

Lactate, acetate, pyruvate and sulfate were measured using an IC (Dionex ICS-5000^+^ DP, Thermo Scientific) equipped with a conductivity-based detector and supplied with MilliQ water (*R* > 18.2 Ω, measured and prepared using an Alto Ultrapure Water System, TripleRed Ltd, Buckinghamshire, UK) for eluent generation. Collected samples were prepared for measurement by centrifuging cell pellets down (5000 rpm for 3.0 min). After separating from the cell pellets, culture supernatants were filtered through a 0.22 µm pore nylon membrane using a Costar Spin-X centrifuge tube filter (Corning Inc., Salt Lake City, USA). The resulting samples were diluted 10 or 100 times (by 10 times dilution series) using MilliQ water (*R* > 18.2 Ω). Each sample (500 ml) was placed into specific IC sampling vials (cat no. 079812, Thermo Fisher Scientific) for analysis, and the IC was run with a sampling size set at 2.5 µl. The autosampler was primed at the beginning of each round of IC analysis according to the equipment manual and using a wash–rinse fluidic cycle. An analytical anion column (Dionex IonPac™ AS11-HC, Thermo Scientific, USA) with 4 µm ion exchange matrix beads was used according to the following separation conditions: 0.38 ml min^−1^ flow rate, 4300 psi pressure and 30°C column temperature. The used eluent was KOH, applied over 37 min with the following gradient profile: 1.5 mM for −7–0 min (pre-run for equilibration), 1.5 mM for 0–8 min (isocratic), increased to 15 mM for 8–18 min, increased to 24 mM for 18–23 min, increased to 60 mM for 23–24 min and stayed at 60 mM for 24–30 min.

### 4.7. DNA isolation, PCR and qPCR

DNeasy Power Soil Kit (QIAGEN, Germany) was used for isolating genomic DNA according to the manufacturer's instructions. This genomic DNA isolation kit was formerly sold by MO BIO as PowerSoil DNA Isolation Kit and used for isolating DNA from bacterial-archaeal co-cultures [28]. Genomic DNA was quantified using NanoDrop spectrophotometer (N60, IMPLEN) and stored at −20 for further analyses.

Specific primers were designed for targeting *dsvA* gene of *Desulfovibrio vulgaris* Hildenborough (IMG gene ID: 637121620), *mtaB* gene of *Methanosarcina barkeri* (IMG gene ID: 637699633) and coenzyme F420 hydrogenase of *Methanococcus maripaludis* (IMG gene ID: 2563556008). The specificity of the developed primers was tested and verified by amplifying the DNAs from monocultures of *Dv*, *Mb* and *Mm* using the polymerase chain reaction (PCR).

The selected primer pairs used in the present study for qPCR detection were Dv_dsvA_1f (5′ → 3′: TTCGTGTCCGACATCAAGCA) and Dv_dsvA_1R (5′ → 3′: GTGGGTTTCACCCTCATCGT) for detecting *Dv* (product length: 135 bp), MB_mtaB_f (5′ → 3′: TGCAAAGAAGACCGGCACTA) and MB_mtaB_r (5′ → 3′: GAGCAGTCCACCACCAATGA) for detecting *Mb* (product length: 85 bp), and Mm_F420_3F (5′ → 3′: TCAACAATACACGGCAACGTA) and Mm_F420_3R (5′ → 3′: GTATCCTTCAGGCGTTCCAA) for detecting *Mm* (product length: 141 bp).

PCR mixtures (in a total volume of 50 µl distilled water) contained 1 µl of 10 mM dNTPs (Bio Lab, USA), 4 µl of 25 mM MgCl_2_ (Promega, USA), 2 µl of forward primer (10 µM), 2 µl of 10 µM reverse primer, 10–20 ng template DNA, 10 µl of GoTaq Flexi Buffer (Promega, USA), 2 µl of 4 mg ml^−1^ bovine serum albumin (Bio Lab, USA) and 0.25 µl of 5 U ml^−1^ GoTaq G2 Flexi polymerase (Promega, USA). PCR mixtures were prepared in bulk volume each time (greater than 500 µl), to minimize preparation errors, and the working volume per sample was 25 µl.

PCR was conducted using a 96-well thermal cycler (Veriti, Applied Biosystems) with the following settings: 95°C for 5 min, 35 cycles of 95°C for 30 s, an annealing temperature of 60°C for 30 s, followed by 72°C for 1 min, and finally 72°C for 10 min. All PCR products were electrophoresed in TAE buffer on 1.0% Hi-Res standard agarose gels (AGTC Bioproducts, UK) with 0.01% GelRed nucleic acid stain (BIOTIUM 10,000X, Hayward CA, USA). DNA band in the gels was visualized by a gel imaging system (U Genius 3, SYNGENE). Agilent Technologies Stratagene Mx3005P real-time PCR system and SYBR Green JumpStart Taq ReadyMix were applied (Sigma-Aldrich, USA) for qPCR analysis.

The genomic sequence lengths excluding plasmids (bp) were retrieved from NCBI for the use in the present work, which are 3570858 bp (NCBI ID: ASM19575v1), 4 533 209 bp (NCBI ID: ASM97002v1), and 1 746 697 bp (NCBI ID: ASM22064v1) for *Dv*, *Mb* and *Mm*, respectively. A standard DNA template for each strain was diluted using sterile water (10-fold dilution series) and tested with the unknown samples in one single qPCR run to generate a standard curve. Each standard sample and replicate in the above experimental design was tested in triplicate under qPCR assay with internal reference dye mode (ROX). The correlation coefficients (*R*^2^) of the standard curves were 0.9987 (*Dv*), 0.9973 (*Mb*) and 0.9999 (*Mm*), and the qPCR efficiencies were 96.1% (*Dv*), 96.8% (*Mb*) and 94.4% (*Mm*).

### 4.8. Mass balance calculations

We performed mass balance calculations based on the assumption that *Methanosarcina barkeri* (*Mb*) and *Desulfovibrio vulgaris* Hildenborough (*Dv*) use only the compounded overall reactions 1–2 and 3–5, shown in table 2, respectively. It is also possible that *Mb* might combine reactions 1 and 2 so to couple acetate reduction with H_2_ oxidation;

$$C_{2}H_{3}O_{2}^{-}+H^{+}+4H_{2}\to 2CH_{4}+2H_{2}O\;\Delta G^{o^{\prime }}=-166.5\;\mathrm{kJ}.\;(\mathrm{reaction}\;6)\;$$

To calculate total methane production in the closed system, we first estimate the amount of acetate and H_2_ available to *Mb*. These compounds can only be produced by *Dv*, through its fermentation pathway, i.e. in reaction 5 from table 1. We thus calculate produced acetate and H_2_ from observed lactate utilization and the stoichiometry of this reaction. The used lactate can be calculated directly from observed lactate at the beginning and end of the cultivation period:

4.1 $$\begin{array}{rl} {\left[\mathrm{Lactate}\right]}_{\mathrm{utilised}} & ={\left[\mathrm{Lactate}\right]}_{\mathrm{initial}}-{\left[\mathrm{Lactate}\right]}_{\mathrm{obs}.\_\mathrm{residual}}\\ {\left[\mathrm{Acetate}\right]}_{\mathrm{prod}.} & ={\left[\mathrm{Lactate}\right]}_{\mathrm{utilised}\;}\\ {\left[H_{2}\right]}_{\mathrm{prod}.} & =2\cdot {\left[\mathrm{Lactate}\right]}_{\mathrm{utilised}} \end{array}\}.$$

The estimated [Acetate]_prod._ and [H_2_]_prod._ need then be combined with the observed residual levels of these compounds in the system, to estimate the levels that were available to *Mb* ([Acetate]*_Mb_* and [H_2_]*_Mb_*):

4.2 $$\begin{array}{rl} {\left[\mathrm{Acetate}\right]}_{Mb} & ={\left[\mathrm{Acetate}\right]}_{\mathrm{prod}.}-{\left[\mathrm{Acetate}\right]}_{\mathrm{obs}\_\mathrm{residual}}\\ {\left[H_{2}\right]}_{Mb} & ={\left[H_{2}\right]}_{\mathrm{prod}.}-{\left[H_{2}\right]}_{\mathrm{obs}\_\mathrm{residual}} \end{array}\}.$$

We can now use these values to calculate the estimated stoichiometric, theoretical methane production ([CH_4_]_calc_) by *Mb*, through reactions 1, 2 and 6. The actual amounts of acetate used in reactions 2 and 6, as well as the actual amounts of H_2_ used in reactions 1 and 6, are unknown. If we assume a full conversion through the three reactions, we would have the following stoichiometric balances:

4.3 $$\begin{array}{rl} \;{\left[\mathrm{Acetate}\right]}_{Mb} & =x_{2}+x_{6}\\ \;\;{\left[H_{2}\;\right]}_{Mb} & =y_{1}+4x_{6}\\ {\left[CH_{4}\right]}_{\mathrm{calc}} & =\frac{y_{1}}{4}+x_{2}+2x_{6} \end{array}\},$$

where *x*_i_ and *y*_i_ denote the amounts of acetate and H_2_ used in reaction *i*, respectively. These three equalities can then be re-arranged to yield the overall theoretical methane production.

4.4 $${\left[CH_{4}\right]}_{\mathrm{calc}}={\left[\mathrm{Acetate}\right]}_{Mb}+\frac{{\left[H_{2}\right]}_{Mb}}{4}.$$

## Supplementary Material

Supplementary file 1

## Data accessibility

All relevant data are presented in the main text and electronic supplementary material. Data are available from the authors in electronic format upon request.
