# Supplementary material for: Increasing sulfate levels show a differential impact on synthetic communities comprising different methanogens and a sulfate reducer
Source: J R Soc Interface. 2019 May 8;16(154):20190129. doi: 10.1098/rsif.2019.0129 (PMC6544901; doi:10.1098/rsif.2019.0129)
Supplement: Supplementary file 1 [file rsif20190129supp1.pdf]

Increasing sulphate levels show a differential impact on synthetic communities comprising different methanogens and a sulphate reducer

Jing Chen[1], Matthew J. Wade[3,4], Jan Dolfing[3], Orkun S. Soyer[*,1,2]

Affiliations: [1] School of Life Sciences, University of Warwick, Coventry, CV4 7AL, UK. [2] Warwick Integrative Synthetic Biology Centre (WISB), University of Warwick, Coventry, CV4 7AL, UK. [3] School of Engineering, Newcastle University, Newcastle, NE1 7RU, UK. [4] School of Mathematics & Statistics, McMaster University, Hamilton, Ontario, L8S 4K1, Canada.

*__Corresponding author:__ Orkun S Soyer, School of Life Sciences, The University of Warwick, Coventry, CV4 7AL, United Kingdom. Phone: +44(0)2476572968. E-mail: O.Soyer@warwick.ac.uk

Supplementary Information

This supplementary text comprises the supplementary figures S1-S4

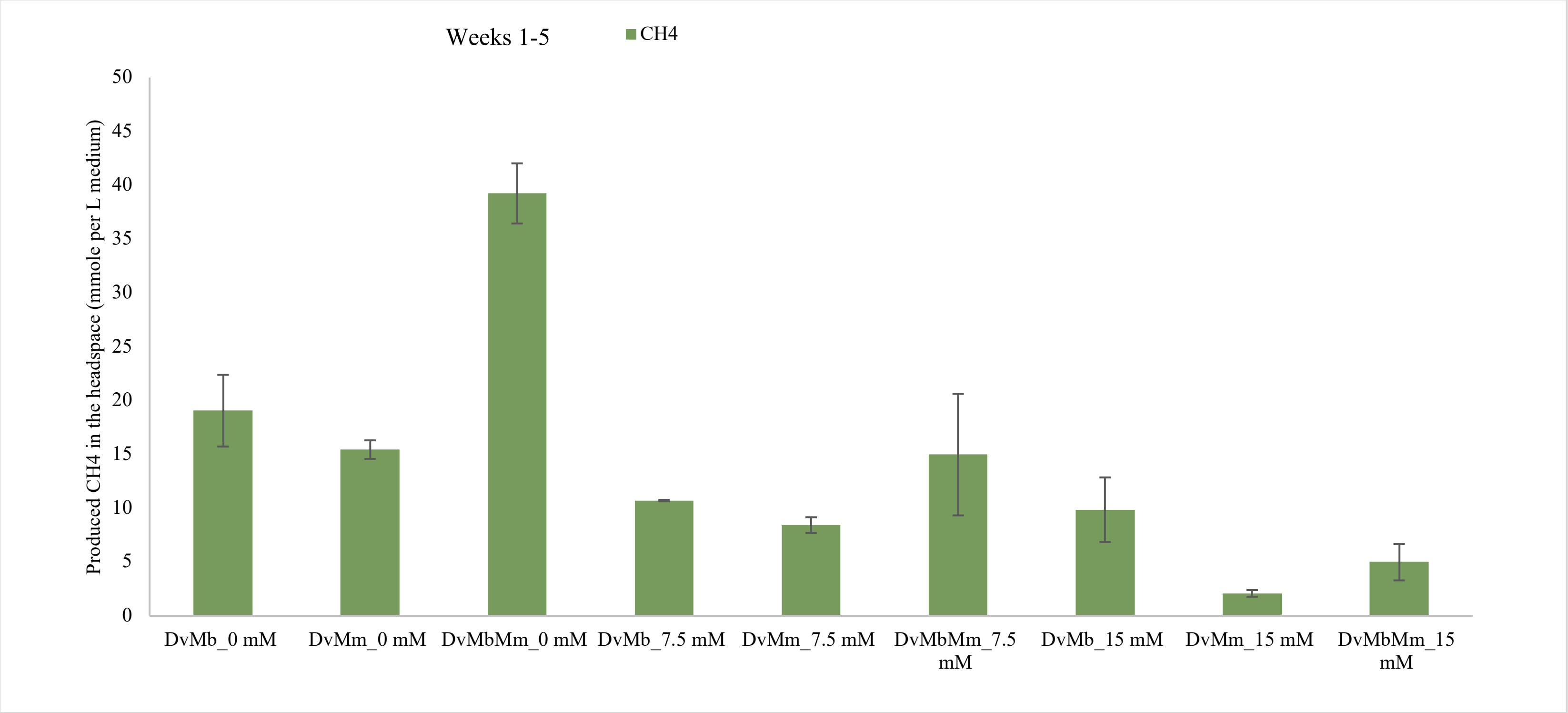

Figure S1. Methane production after 5 weeks' cultivation. Results of different co- and tri-cultures of species *Desulfovibrio vulgaris* (*Dv*), *Methanococcus maripaludis* (*Mm*) and *Methanosarcina barkeri* (*Mb*), as indicated on the x-axis. The indicated mM concentration refers to sulphate amounts in the initial media. Results and error bars are those from three replicates, except for DvMbMm_7.5 mM, DvMb_15 mM, DvMm_15 mM, and DvMbMm_15 mM cases, which are based on two replicates only. Data were calculated using methane fraction measured by Micro-GC and pressure change before and after 5 weeks' incubation monitored by needle gas gauge. Results from 5 mL test tube cultures are used to extrapolate to 1 L culture output, so to achieve a better comparison of gas and organic acid data.

(a)

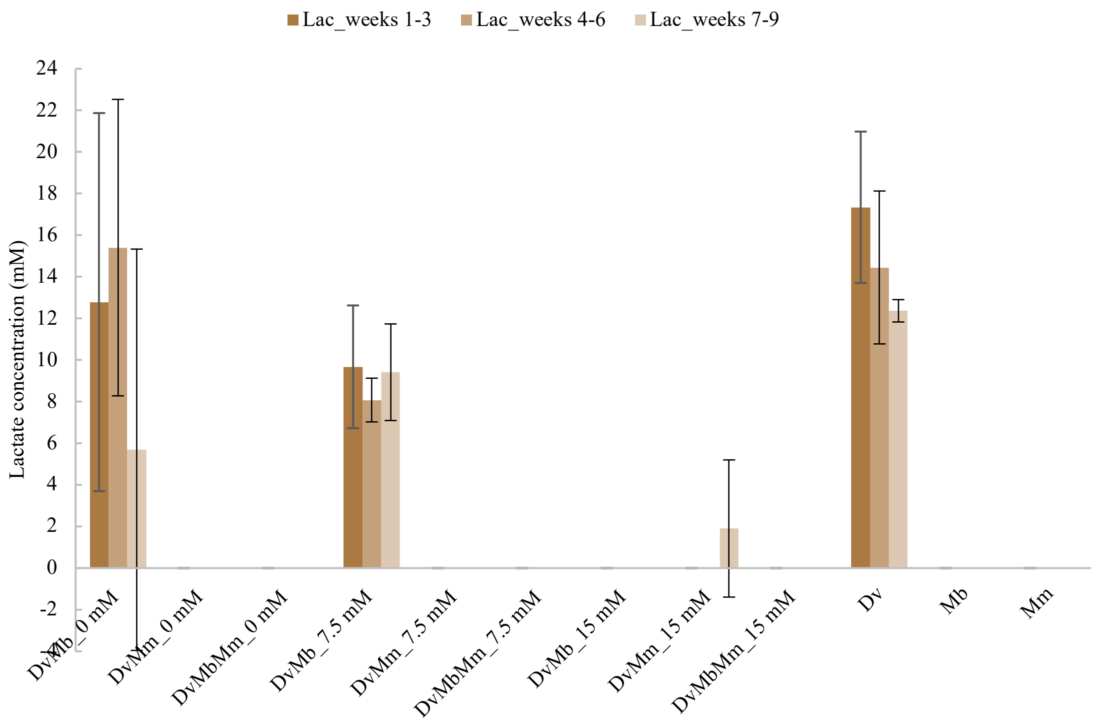

(b)

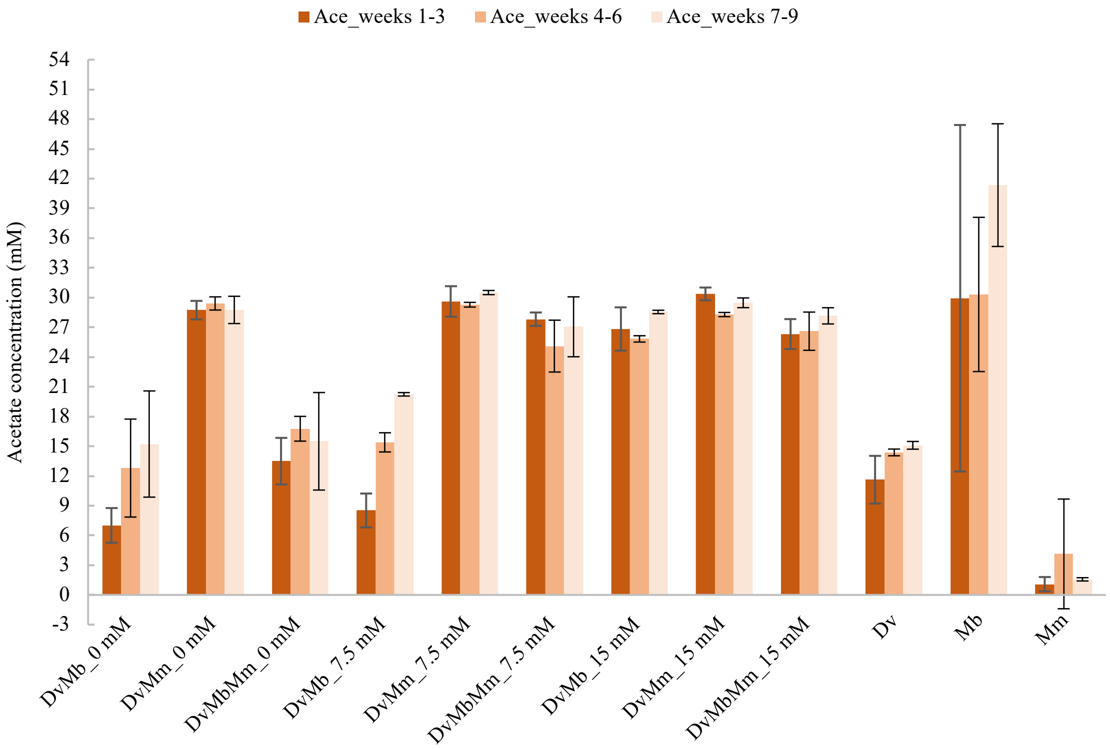

Figure S2. Lactate **(a)** and acetate **(b)** concentrations after 3 weeks' cultivation. Results of different mono-, co- and tri-cultures of species *Desulfovibrio vulgaris* (*Dv*), *Methanococcus maripaludis* (*Mm*) and *Methanosarcina barkeri* (*Mb*), as indicated on the x-axis. The indicated mM concentration refers to sulphate amounts in the initial media. The mono-cultures were grown in the same basal media as co- and tri-cultures, but with additional

supplements to allow growth; *Dv* monocultures were grown in medium using 30 mM/L Na-lactate as carbon source with 10 mM/L Na_2SO_4; *Mb* monocultures were grown in medium using 100 mM/L Na-acetate as carbon source; *Mm* monocultures were grown in medium using 10 mM/L Na-pyruvate with 80% H_2-20% CO_2 headspace. All results are from three replicates.

(a)

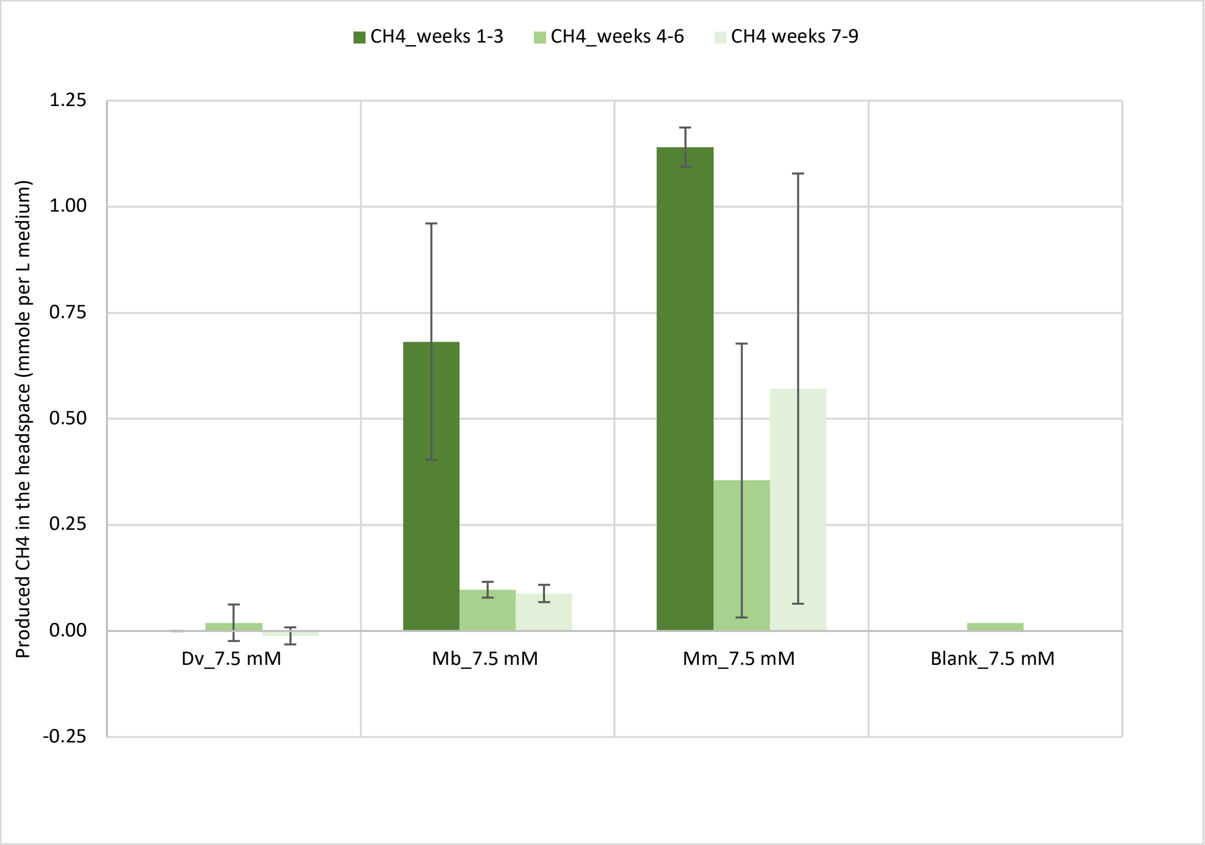

(b)

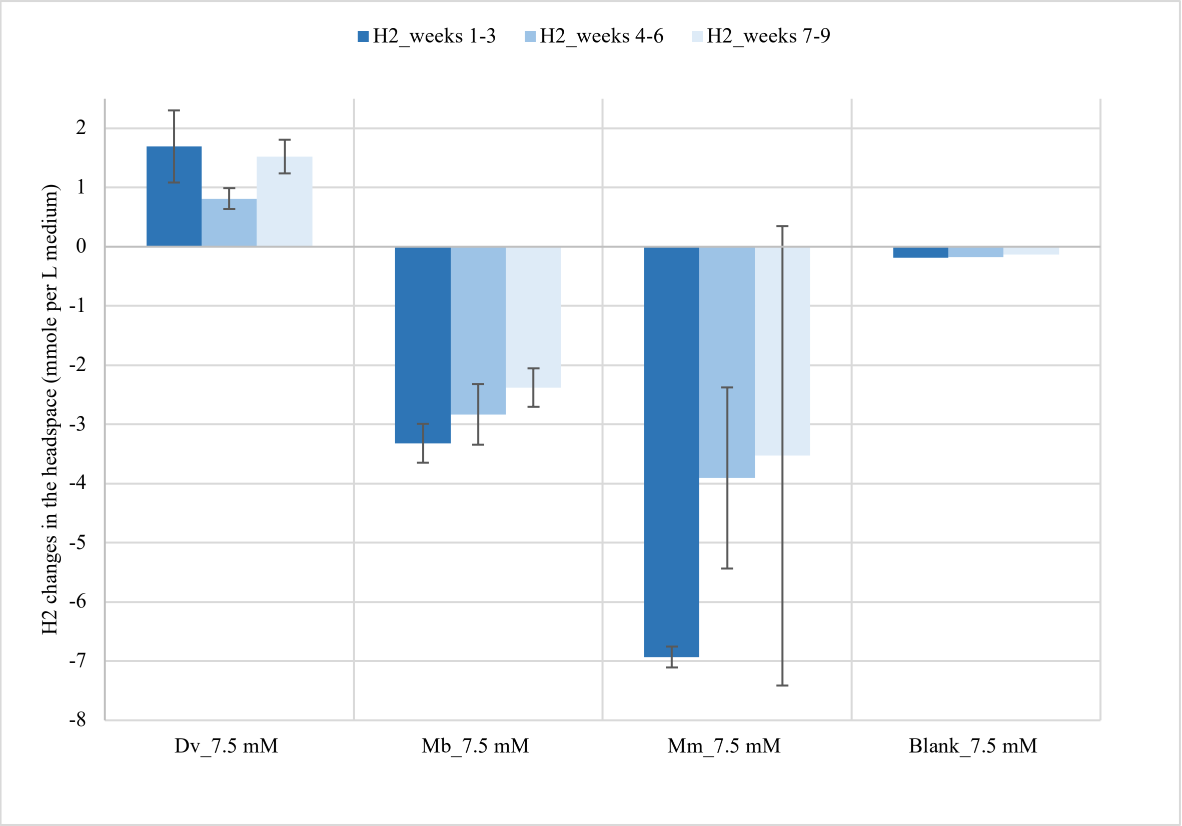

Figure S3. Methane production **(a)** and hydrogen change **(b)**, as measured in the headspace of monocultures for species *Desulfovibrio vulgaris* (*Dv*), *Methanococcus maripaludis* (*Mm*) and *Methanosarcina barkeri* (*Mb*). The mono-cultures were grown in the same media as co-

and tri-cultures and using 30 mM Na-Lactate as carbon source and with 7.5 mM/L sulphate addition (so to serve as a negative control for methane production; see *Methods*). Data were calculated using methane and hydrogen fractions measured by Micro-GC and pressure change before and after 3 weeks' incubation monitored by needle gas gauge. Results from 5 mL test tube cultures are used to extrapolate to 1 L culture output, so to achieve a better comparison of gas and organic acid data. All results are from three replicates.

(a)

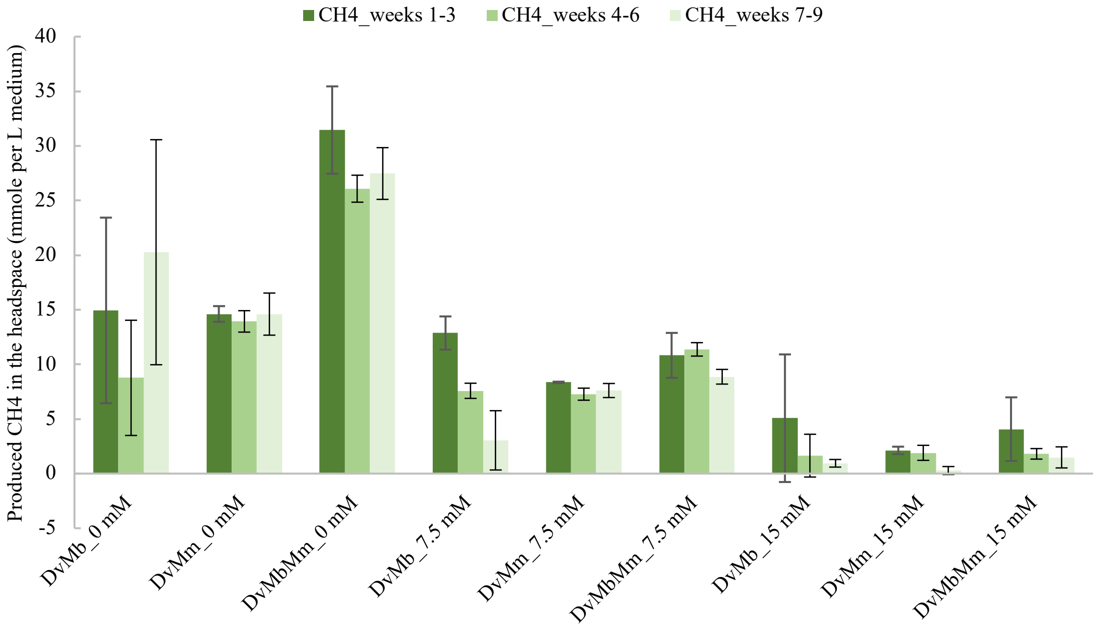
CH4_weeks 1-3
CH4_weeks 4-6
CH4_weeks 7-9
Produced CH4 in the headspace (mmole per L medium)
40
35
30
25
20
15
10
5
0
-5
DvMb_0 mM
DvMm_0 mM
DvMbMm_0 mM
DvMb_7.5 mM
DvMm_7.5 mM
DvMbMm_7.5 mM
DvMb_15 mM
DvMm_15 mM
DvMbMm_15 mM

(b)

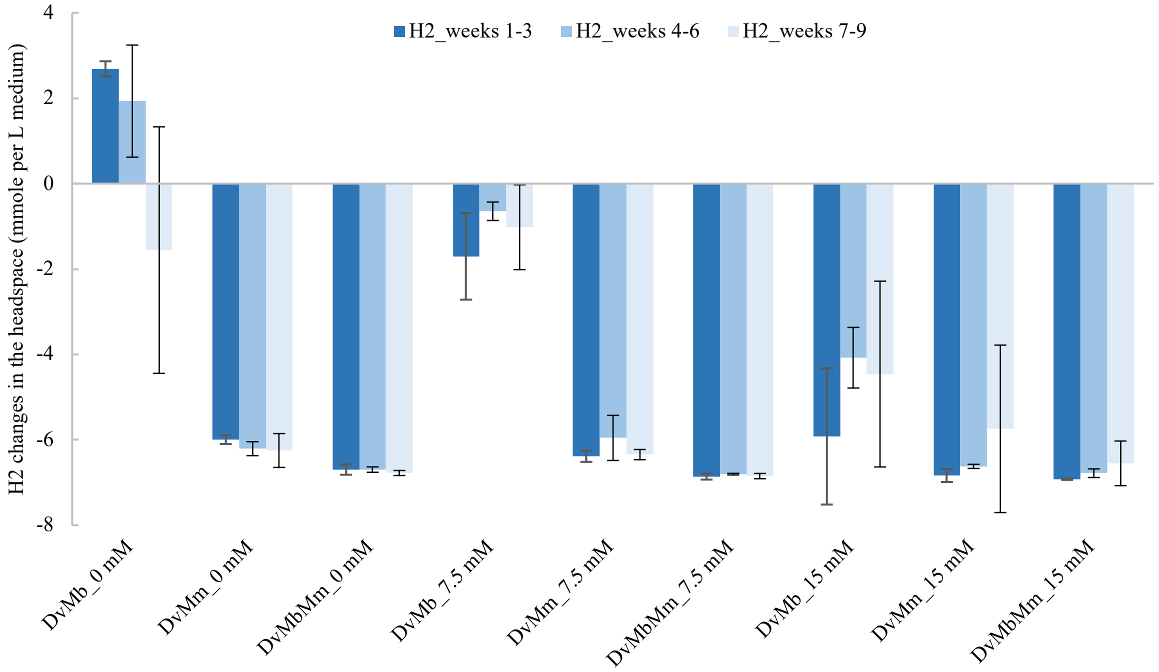
H2_weeks 1-3
H2_weeks 4-6
H2_weeks 7-9
H2 changes in the headspace (mmole per L medium)
4
2
0
-2
-4
-6
-8
DvMb_0 mM
DvMm_0 mM
DvMbMm_0 mM
DvMb_7.5 mM
DvMm_7.5 mM
DvMbMm_7.5 mM
DvMb_15 mM
DvMm_15 mM
DvMbMm_15 mM

(c)

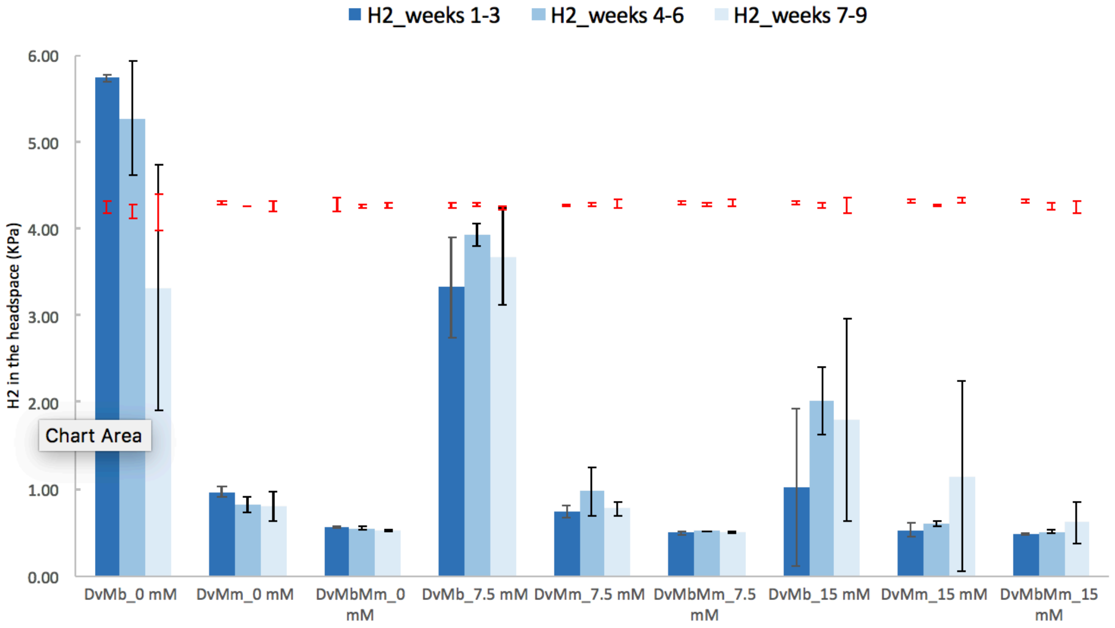

Figure S4. End-point methane production **(a),** hydrogen change **(b)**, and absolute hydrogen levels **(c)** as measured in the headspace of co- and tri-cultures of species *Desulfovibrio vulgaris* (*Dv*), *Methanococcus maripaludis* (*Mm*) and *Methanosarcina barkeri* (*Mb*), as indicated on the x-axis. The indicated mM concentration refers to sulphate amounts in the initial media. Data were calculated using methane and hydrogen fractions measured by Micro-GC and pressure change before and after 3 weeks' incubation monitored by needle gas gauge. Results from 5 mL test tube cultures are used to extrapolate to 1 L culture output, so to achieve a better comparison of gas and organic acid data. Red error bars in Fig. S5C refer to the initial hydrogen levels in the headspace of each individual tube and the deviation among these three replicates. The initial H_2 in the tube headspace was 3.14% ±0.03 (in the unit of mmole per L medium correlated to 1 atm: 7.46 ±0.07). All results are from three replicates.
